# Validity and Reliability of the Iranian Version of the Short Form Social Well Being Scale in a General Urban Population

**Published:** 2019-08

**Authors:** Zeinab SHAYEGHIAN, Parisa AMIRI, Golnaz VAHEDI-NOTASH, Mehrdad KARIMI, Fereidoun AZIZI

**Affiliations:** 1. Research Center for Social Determinants of Health, Research Institute for Endocrine Sciences, Shahid Beheshti University of Medical Sciences, Tehran, Iran; 2. Endocrine Research Center, Research Institute for Endocrine Sciences, Shahid Beheshti University of Medical Sciences, Tehran, Iran

**Keywords:** Psychometric properties, Social well-being, Validity, Reliability, Iran

## Abstract

**Background:**

We aimed to investigate the reliability and validity of the Iranian version of the short form social well-being scale.

**Methods:**

After linguistic validation, the Iranian version of social well-being scale was completed by 715 participants, aged ≥18 yr between Jan and Sep 2015. Concurrent validity was examined by calculating the Pearson’s correlation coefficient between the dimensions of social well-being and social support. Internal consistency, Cronbach’s alpha coefficient, confirmatory and explanatory factor analyses were also examined.

**Results:**

The internal consistency as measured by Cronbach’s alpha coefficients was 0.72 for total score. No ceiling and floor effect was found in total score or any of the subscales. The concurrent correlation coefficients between social well-being and social support ranged from good to excellent agreement. Exploratory factor analysis supported mainly comparable results with the original US English dialect version. The results of the confirmatory factor analysis for 5-factor models indicated acceptable fit for the proposed research models.

**Conclusion:**

The findings support the initial reliability and validity of the Iranian version of the social well-being scale in the research and community settings in Iran.

## Introduction

Well-being is a dynamic concept that includes subjective, social, and psychological dimensions as well as cognitive and affective components (1, 2). The cognitive component refers to the life satisfaction and subjective evaluation of people about their life circumstances and the effective component refers to the positive and negative emotional effects and the balance of these states experienced by individuals over time (3). Generally, the studies of well-being could be divided into three aspects: Hedonic well-being, eudaimonic well-being and social well-being (4, 5).

Hedonic well-being is about pleasure and happiness (4, 6, 7). There are many ways for evaluating pleasure but the most recent hedonic psychology has used assessment of subjective well-being. Subjective well-being is a construct formed by frequently positive feelings such as love and life satisfaction, and rarely by negative feelings such as sadness (6, 8). Eudaimonic well-being reflects traits concerned with personal growth, self-acceptance and purposeful life (9) that is related to the development of a person’s best potentials (10). Social well-being is an explanation of people’s perception and experience of being in a good situation, satisfaction with the structure, and social interaction (11–14).

According WHO definition of health, it is a state of complete physical, mental and social well-being and not merely the absence of disease or infirmity, social well-being has been investigated more in public health research during the last decades (15). While the main focus of hedonic and eudaimonic well-being are on personal belief and experiences, social well-being concentrates on public side of life where life experiences and challenges that could affect individuals’ health occur (5, 16). Despite current focus being on social well-being, limited studies have documented a theoretical basis or an objectively clear definition of this concept (17). Conventionally, social well-being was often considered as social circumstances, social economic status and life circumstances and has been consequently measured by related indices including GDP or GNP and the rate of poverty and inequality (17–19). To the best of our knowledge, among the theory-based instruments previously developed, the social well-being questionnaire introduced by Keyes (17) seems to be a well-known instrument validated and applied in various countries in its complete and short forms (5, 20, 21). The short form version of this instrument includes 15 items, used to measure and evaluate the individual’s perceptions of his/ her integration into society and the coherence of social events and of his/ her acceptance by other people and his/ her contribution to society (11).

Although the Iranian version of the social well-being questionnaire has been previously applied for a population of university students (20), the validity and reliability of this questionnaire have not been examined in general Iranian populations with different socio-demographic characteristics. We for the first time aimed to examine validity and reliability of the Iranian version of short-form social well-being scale in a general population of Iranian adults, residing in Tehran.

## Methods

### Subjects and design

This study has been conducted in the framework of the Tehran Lipid and Glucose Study (TLGS). Details of the rationale and design of the TLGS has been published elsewhere (22). A multistage stratified cluster random sampling technique was used to select the TLGS participants from the urban district 13 of Tehran. In the current study, data for 715 individuals, aged ≥ 18 yr (59% women) participated in the TLGS between Jan and Sep 2015 and completed social well-being questionnaire has been analyzed. Participants were interviewed by a trained interviewer to collect data on social well-being and other socio-demographic data.

All participants gave written informed consent. The study was approved by the Ethics Committee of the Research Institute for Endocrine Sciences, Shahid Beheshti University of Medical Sciences.

### Measures

#### Demographic questionnaire:

Demographic data on gender, age, marital status, level of education and employment status were obtained from TLGS database.

#### The Multidimensional scale of perceived social support (MSPSS):

The MSPSS is a multidimensional 12-item self-report measure which has been developed (23). Psychometric properties of the scale are well established abroad, with coefficients of internal consistency of 0.85 to 0.91 and test-retest reliabilities of 0.72 to 0.85 (24). Moreover, the Iranian version of MSPSS has been already validated with acceptable internal consistencies in both sick and healthy populations (25).

#### The short-form social well-being questionnaire:

The short-form social well-being questionnaire developed by Keyes (26), contains 5 dimensions, each of which is measured by 3 questions. Participants should answer these questions using the following options: 1=Agree strongly to 7= Disagree strongly. Negatively worded items were reverse coded before any analyses. The social well-being dimensions consist of Social integration, social coherence, social acceptance, social contribution and social actualization (26, 27). Social integration is the sense of belonging to society and the quality of individuals’ relationship with communities and society (26, 28, 29). Social coherence is the perception of the quality organization in a social world and related aspects (11, 28). Social acceptance is having favorable views of other people and feeling comfortable with them; this generalized category is an interpretation of society through the character and qualities of other people (26, 28). Social contribution is about feeling important, efficacious and valuable for society as a member of it (11, 26, 28). Social actualization is believing that the institutions and individuals are helping to reach their potentials in society (26, 28).

### Procedure

In the present study after getting permission and obtaining the previously translated questionnaire from Dr. Keyes, the Persian version of short-form social well-being questionnaire was assessed item by item, by a psychologist and a sociologist. To ensure ability of individuals to read and understand, 40 people with different ages and educational levels completed the translated questionnaire through a pilot study. All ambiguities of translated version were resolved and it was prepared to use in the current study.

### Statistical Analysis

Continuous variables were described by mean ± SD; categorical level variables were described by frequencies and percent. For the construct validity assessment, Exploratory Factor Analysis (EFA) was conducted on 50% randomly sampled data (358 cases) to detect social-well-being subscales. The number of factors was determined using the extraction method of principal component analysis. Varimax with Kaiser Normalization was considered to rotate the factors. Confirmatory Factor Analysis (CFA) was conducted on the remaining 50% randomly sampled data (357 cases) to assess and confirm the statistical construct validity of the Iranian version of the social-well-being questionnaire and its replicability (30). The hypothesized model used for relationships between latent constructs and their identified items was based on EFA results and previous analytic research in the short form of Keyes’ questionnaire (26).

Cronbach’s coefficient alpha coefficients have been calculated to determine the internal consistency reliability of social well-being and its subscales (31). Concurrent validity refers to the degree of correlation between evaluated construct and other measures of the same construct measured at the same time (32). Concurrent validity of the Persian version of the social well-being scale was evaluated using the Pearson correlation and Bland-Altman agreement plot between this scale and the MSPSS which was measured at the same time on the same subjects to obtain validity (32).

For known-group validity assessment, the mean of social well-being subscales and its total score were compared between individuals of different ages and educational levels, using one way ANOVA. Tukey Post Hoc test was used for pairwise comparisons between groups, which were significantly different. If the consistency of variances between groups did not hold, Welch correction and Tamhane’s T2 were conducted for group comparison and pairwise evaluations, respectively. IBM SPSS Statistics software Version 20 & AMOS version 20 was used for data descriptions and statistical analysis (33).

## Results

Participants were 715 individuals (59% women), aged ≥ 20, who participated in the TLGS study from 2014–2015; 74.4% were married and 34.3% were employed. Other features of participants are shown in Table 1. The internal consistency of the scale as measured by Cronbach’s alpha coefficients and the mean±SD for total and each of the subscale scores have been reported in Table 2.

**Table 1:** Demographic characteristics of study participants (N=715)

***Variable***	***Scale***	***Frequency***	***Percent (%)***
Gender	Men	293	41
	Women	422	59
Age (yr)	≤20	17	2.4
	20–40	202	28.3
	40–60	289	40.4
	>60	207	29
Marital status	Single	120	16.8
	Married	531	74.4
	Widowed/divorced	63	8.8
Level of education	Primary	122	17.6
	Secondary	378	54.5
	Higher	194	28
Employment status	Employed	244	34.3
	Unemployed with income	34	4.8
	Unemployed	434	61

**Table 2:** Means, standard deviations, and Cronbach’s alpha for the Iranian version of Keyes scale

***Variable***	***Mean***	***SD***	***Percent Floor***	***Percent Ceiling***	***Minimum***	***Maximum***	***Cronbach’s alpha***
Total score	65.62	13.25	.3	.3	25	98	.72
Social integration	14.63	3.99	1.5	4.2	3	21	.33
Social coherence	13.69	4.28	2.4	3.6	3	21	.44
Social acceptance	8.78	2.80	1.7	4.2	2	14	.40
Social contribution	14.18	4.95	4.1	6.4	3	21	.66
Social actualization	14.33	4.83	2.7	9.9	3	21	.62

Cronbach’s alpha in subscales ranged between 0.33 (social integration) to 0.66 (social contribution). The internal consistency of the total scale with deleted item 7 was 0.72. Social integration and acceptance had the highest (14.63±3.99) and lowest (8.78±2.80) means respectively. No ceiling or floor effect was found in the total score or any subscale.

**Fig. 1:**
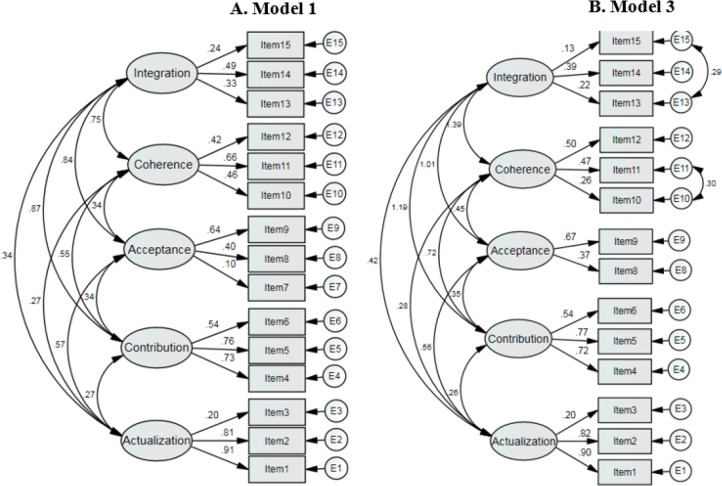
Measurement models of Social Well-Being Scale with standardized estimations Model A: The original conceptual measurement model of social well-being constructs Model B: Covariance between error variances of items 13&15 and items10&11were considered and item 7 was removed

The results of EFA have been shown in Table 3. The determinant index of 0.095 indicated no collinearity problem among observed variables. The Kaiser-Mayer-Olkin (KMO) of 0.70 confirmed sample size adequacy and Bartlett’s Test of Sphericity (Chi-Square = 823.21, DF=105, *P*<0.001) indicated that this assumption would hold. Eigenvalue greater than one was considered to detect the number of factors. Results revealed five distinct factors, exploring 55.40% of total variance (Table 3). Minimum and maximum initial eigenvalues were 2.92 and 1.05 for the first and the fifth factors respectively. All items were loaded above the acceptable threshold (lambda > 0.40), except item 7 from factor 3; there is no cross loading on multiple factors. Regarding the 3- step hierarchy CFA, as the first step, the original conceptual measurement model was evaluated (Table 4). Except for item 7 of the “acceptance” construct, all factor loadings had significant explanations (*P* < 0.001) (Fig. 1 A). As the second step (Model 2), two covariance structures were added to the original model including the covariance between error variances of items 13 and 15 (correlation=0.29, *P*<0.001) and the covariance between error variances of items 10 and 11 (correlation=0.30, *P*<0.001) (model 2).

**Table 3:** Exploratory Factor Analysis results for the Iranian version of Social well-being scale

***Items***	***Factor 1***	***Factor 2***	***Factor 3***	***Factor 4***	***Factor 5***
Social Actualization					
1. Society isn’t improving for people like me	.876				
2. Society has stopped making progress	.865				
3. You don’t think social institutions like law and government make your life better	.644		.112	.106	
Social Contribution					
4. My daily activities do not produce anything worthwhile for my community		.788			−.184
5. I have nothing important to contribute to society	.115	.740	−.169	.182	
6. I have something valuable to give to the world		.582			.174
Social Acceptance					
7. People who do a favor expect nothing in return		−.154	.674	−.235	−.118
8. I believe that people are kind	.170		.528	.206	.151
9. People do not care about other people’s problems	.242	.166	.527		.263
Social Coherence					
10. I find it easy to predict what will happen next in society				.840	
11. I cannot make sense of what’s going on in the world	.129	.198		.683	.142
12. The world is too complex for me	.112	.367	.135	.401	.184
Social Integration					
13. My community is a source of comfort	.118	−.128		.105	.707
14. I don’t feel I belong to anything I’d call a community	.117	.383	−.162		.610
15. I feel close to other people in my community	−.250	.160	.466	.131	.472
Explained Variance (%)	14.34	12.87	10.23	9.14	8.81

Total explained variance: 55.40%

Factor loadings above 0.10 displayed and above 0.40 were bolded.

**Table 4:** Fit indices of measurement models (CFA) for social well-being constructs

***Model***	***X^2^***	***DF***	***X^2^/DF***	***RMSEA***	***SRMR***	***CFI***	***GFI***	***IFI***	***TLI***	***AIC***
1	250.68	80	3.13	0.077	0.08	0.83	0.90	0.83	0.78	330.68
2	201.55	78	2.58	0.067	0.07	0.88	0.92	0.88	0.84	285.55
3	150.92	65	2.32	0.060	0.06	0.91	0.94	0.91	0.90	230.92

X^2^: Chi-Square value; DF: Degrees of Freedom; RMSEA: Root Mean Square Error of Approximation; NFA: Normed Fit Index; CFA: Comparative Fit Index; IFI: Incremental Fit Index; SRMR: Standardized Root Mean Square Residual; GFI: Goodness of Fit Index; AIC: Akaike Information Criteria

Model 1: The original conceptual measurement model of social well-being constructs

Model 2: Covariance between error variances of items 13&15 and items10&11were considered

Model 3: Covariance between error variances of items 13&15 and items10&11were considered and item 7 was removed

Chi-square differences between the two CFA models illustrate significant improvement in this statistical index (Δχ^2^ = 49.13, DF=2, *P*<0.001) and the other fit indices were also slightly better than model 1. Again, all of the factor loadings had significant explanations (*P*<0.001), except item 7 of the “acceptance” construct. Hence, in the third model, after removing item 7 of the “acceptance” construct, compared to the second model more significant improvement occurred in the chi-square differences (Δχ^2^ = 50.63, DF=13, *P*<0.001) and the other fitting indices (Table 4 & Fig. 1.B). As a result, the fit indices of the Iranian version of the social well-being questionnaire with 5 factor and 14 items were acceptable. Correlations between 5 factors which have been demonstrated by two-sided pathways were significant for all of the CFA models (*P*<0.05) except for correlation between integration and actualization (*P*=0.054).

**Fig. 2:**
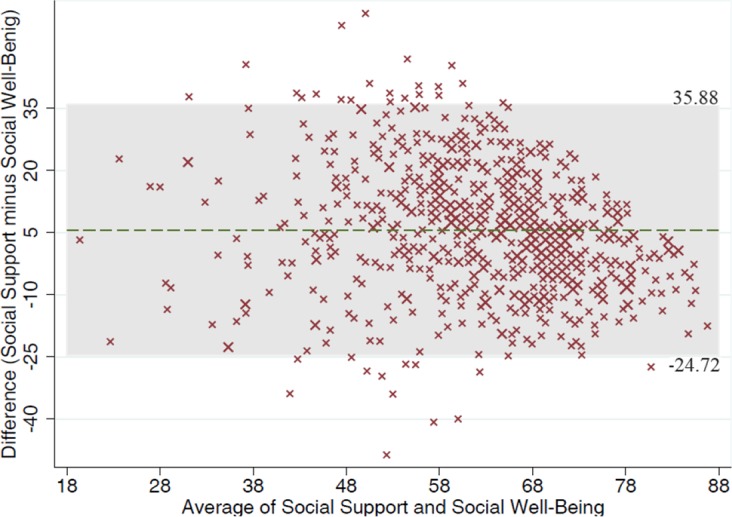
The Bland-Altman agreement plot between social support and social well-being scales

The results of Pearson’s correlation displayed that all five components of the Iranian version of short-form social well-being questionnaire were significantly related to MSPSS and its subscales (Table 5). The Intra-Class Correlation Coefficient (ICC) between these two questionnaires, 0.54 with 95% CI of (0.47, 0.61), indicate significant consistency or reproducibility of the two scales. Accordingly, the Bland-Altman agreement plot between social support and social well-being showed the 95% confidence interval of agreement between two scales was in the range of (−24.72–35.88) (Fig. 2), confirming no significant differences between them. The average range of two scales was between 19.39 and 86.77. Only 35 cases (4.90%) were outside the limit of agreement and were considered as outliers.

**Table 5:** Pearson Correlation Coefficients among social support and social well-being subscales

***Variable***	***Social support***	***Family***	***Friends***	***Others***	***Social well-being***	***Integration***	***Coherence***	***Acceptance***	***Contribution***	***Actualization***
1. Social support	1									
1.1. Family	.746^**^	1								
1.2. Friends	.711^**^	.209^**^	1							
1.3. Others	.801^**^	.574^**^	.281^**^	1						
2. Social well-being	.368^**^	.262^**^	.300^**^	.260^**^	1					
2.1. Social integration	.431^**^	.257^**^	.420^**^	.270^**^	.637^**^	1				
2.2. Social coherence	.155^**^	.141^**^	.091^*^	.123^**^	.662^**^	.292^**^	1			
2.3. Social acceptance	.200^**^	.147^**^	.173^**^	.127^**^	.585^**^	.298^**^	.236^**^	1		
2.4. Social contribution	.212^**^	.157^**^	.176^**^	.142^**^	.660^**^	.327^**^	.324^**^	.171^**^	1	
2.5. Social actualization	.182^**^	.134^**^	.115^**^	.163^**^	.612^**^	.154^**^	.216^**^	.392^**^	.128^**^	1

** Correlation is significant at the 0.01 level (2-tailed). //

* Correlation is significant at the 0.05 level (2-tailed).

There were statistically significant differences between different age groups for social integration, social contribution, and social actualization. Increase in age level raised the mean of actualization. These results illustrate significant mean differences in social well-being total scores and some of its subscales in those with different educational levels. Except for the acceptance and actualization subscales, people with higher educational levels had significantly better social well-being scores. In addition, the means of the integration, contribution and actualization subscales scores differed significantly among different age groups (Table 6).

**Table 6:** Mean and standard deviation of Social Well-Being subscales among baseline characteristics: A Sub-group analysis

***Variable***		***Total Score***			***Social Integration***			***Social Coherence***			***Social Acceptance***			***Social Contribution***			***Social Actualization***		
		***Mean***	***SD***	***Sig.***	***Mean***	***SD***	***Sig.***	***Mean***	***SD***	***Sig.***	***Mean***	***SD***	***Sig.***	***Mean***	***SD***	***Sig.***	***Mean***	***SD***	***Sig.***
Age(yr)	<=20	62.64	12.56	.213	14.94	3.40	.044	13.94	4.32	.909	7.88	3.12	.238	14	3.77	.000	11.88	4.10	.027
	21–40	66.73	12.31		14.91	3.74		13.51	4.31		8.54	2.57		15.68	3.84		14.05	4.47	
	41–60	65.97	13.58		14.89	3.93		13.79	4.32		8.87	2.88		14.16	5.01		14.25	5.11	
	> 60	64.31	13.65		13.97	4.31		13.71	4.24		8.95	2.89		12.76	5.47		14.90	4.76	
Education	Primary	62.80	14.18	.000	13.69	3.84	.002	13.18	4.61	.006	9.05	2.92	.427	11.89	5.51	.000	14.97	4.62	.235
	Secondary	64.87	12.56		14.59	4.00		13.47	4.20		8.67	2.80		13.86	4.72		14.26	4.88	
	Higher	69.22	13.57		15.31	4.22		14.53	4.22		8.79	2.73		16.53	3.85		14.04	4.91	

## Discussion

This study examined the validity and reliability of the Iranian version of the short form social well-being questionnaire in a general urban population. Our results support the initial reliability and validity of the Iranian version of the social well-being scale. Cronbach’s alpha coefficient to test reliability was acceptable for the total score (0.71). This level of internal consistency is comparable to the original version and other translated versions of the questionnaire (5, 26). The range of Cronbach’s alpha coefficients for the original and Iranian versions of the questionnaire were 0.41 (acceptance) −0.73 (integration) and 0.34 (social integration) −0.66 (social contribution) respectively. Hence compared to the original questionnaire, the Iranian version showed lower level of internal consistency in three subscales including integration, coherence and acceptance. There were no floor and ceiling effects in total or any of subscales.

The results of the exploratory factor analysis suggested a five-factor structure consistent with the original scale (26). Except for item 7 in the third factor (acceptance subscale) all other items were loaded in the hypothesized scale, consistent with the original version. This item had the same status in both the original and also Chinese versions of the social well-being scale (5, 26). In addition, this was probably due to negative meaning of the item mentioned that confused participant. Keyes justified the low acceptance value with the added new item for balancing the positive and negative item (26). Regarding which, combination of positive and negative items decreased the questionnaire homogeneity (5). The current results of the confirmatory factor analysis revealed acceptable fit indices for the Iranian version of social well-being scale as the original and the Chinese versions (5, 26).

Due to closeness of two concepts of social support and social well-being, in this study, the correlation between the Iranian version of the short form social well-being questionnaire and MSPSS has been assessed. The psychometric properties of the Iranian version of MSPSS has been previously investigated and confirmed (23, 25). Social well-being was examined with two elements, social adjustment and social support (18). There is increasing evidence showing common beliefs regarding social support as one of the components of social well-being (17, 34); hence it was reasonable to hypothesize a logical agreement between the social support and social well-being scores. In this study, the positive correlations of subscales of social well-being with social support were significant and supported the validity of the Iranian version of the questionnaire. The correlation coefficients between social well-being and social support (and their subscales) indicate good to excellent validity. As a result of agreement analysis between two scales, no existence of any systematic difference, including fixed bias between the measurements of two scales and two scales may be used interchangeably.

In the Keyes study, age and education were chosen as elements that affected social well-being and both showed significant differences. In this study, there were significant differences between different age groups in the three subscales. While the mean differences in age group were not considerable, we found lower social integration in the old (>60) and middle-aged (41–60 yr) person. In addition, people in 21–60 yr old had higher social contribution those <20 and >60 yr. This a result also reported in previous investigations (11, 26). On the other hand, our findings showed significant differences in total score and all subscales of social well-being, except social actualization and social acceptance in terms of education status, people with high degrees had more social well-being than others, a finding is consistent with another study (26). The replication of age and educational differences in social well-being illustrated the role of social forces in shaping social well-being (26). Moreover, with aging and its impact on a person’s perception, the individual’s social well-being naturally change during the lifetime (17).

This study is one of the first efforts to assess psychometric properties of the Iranian version of short-form of social well-being scale in a large general urban population. However, explaining some limitations of the current study is noteworthy. We could not conduct test-retest reliability to examine reproducibility of the initial results. Furthermore, the study population was just from Tehran, the capital city of Iran, which could decrease the level of generalizability of our findings.

## Conclusion

The Iranian version of the social well-being scale has psychometric properties similar to those of the original version of the scale. Our findings confirmed the initial reliability and validity of the Iranian version of social well-being.

## Ethical considerations

Ethical issues (Including plagiarism, informed consent, misconduct, data fabrication and/or falsification, double publication and/or submission, redundancy, etc.) have been completely observed by the authors.
